# Strikes of physicians and other health care workers in sub-Saharan African countries: a systematic review

**DOI:** 10.3389/fpubh.2024.1209201

**Published:** 2024-05-30

**Authors:** Alexandre Lourenço Jaime Manguele, Mohsin Sidat, Paulo Ferrinho, António Jorge Rodrigues Cabral, Isabel Craveiro

**Affiliations:** ^1^Instituto Superior de Ciências de Saúde, Maputo, Mozambique; ^2^Global Health and Tropical Medicine, GHTM, LA-REAL, Instituto de Higiene e Medicina Tropical, IHMT, Universidade NOVA de Lisboa, Lisboa, Portugal; ^3^Faculdade de Medicina, Universidade Eduardo Mondlane, Maputo, Mozambique

**Keywords:** strikes, industrial actions, health care workers, physicians, nurses, sub-Saharan Africa, systematic review

## Abstract

**Introduction:**

Strikes in the health sector have been of growing concern, given their disruptive nature, negatively impacting the provision of health care and jeopardizing the well-being of patients. This study aims to identify the main actors, the reasons behind industrial actions protests, strikes and lockouts (IAPSL) in sub-Saharan African countries and their impact on health care workers (specifically doctors) and health services, as well as to identify the main strategies adopted to reduce their impact on healthcare services.

**Methods:**

Studies published between January 2000 and December 2021 and archived in MEDLINE, Google Scholar, Scopus, ProQuest, and Science Direct were included. Quantitative, observational (i.e., cohort, case-control, cross-sectional, and ecological) and experimental studies, as well as mixed methods, quasi-experimental, and qualitative studies were eligible

**Results:**

A total of 5521 studies were identified and after eliminating duplicates, applying the inclusion criteria, and assessing the risk of bias, a total of 11 studies were included in the review. Nurses and doctors are the actors most commonly involved in strikes. The main causes of strikes were salary claims and poor working conditions. The main strategies adopted to mitigate the strike consequences were to restrict services and prioritize emergency and chronic care, greater cooperation with the private sector and rearrange tasks of the available staff. The strikes led to a reduction in hospitalizations and in the number of women giving birth in health units, an increase in maternal and child morbidities and delays in the immunization process. Increased mortality was only reported in faith-based hospitals.

**Discussion:**

This evidence can assist decision-makers in developing strategies and interventions to address IAPSL by health care workers, contributing to strengthen the health system. Strikes in the health sector disrupt healthcare services provision and compromise the well-being of patients, especially the most disadvantaged, with consequences that may be difficult to overcome ever. The potential health impacts of strikes highlights the importance of their prevention or timely resolution through regulation and negotiations to balance the rights of health care workers and the rights of patients.

**Systematic review registration:**

https://www.crd.york.ac.uk/PROSPERO/display_record.php?RecordID=334173, identifier CRD42022334173.

## 1 Introduction

A strike is defined as a temporary stoppage of work by a group of employees to express a complaint or enforce a demand (1). Strikes can range from a simple interruption of the workday (for a few hours) or the reduction of non-critical services, to the complete stoppage of work as a last resort (2).

Strikes in the health sector have been of growing concern, given their disruptive nature, negatively impacting the provision of health care and jeopardizing the well-being of patients (1, 3). The effects of the outage of health services can vary based on the organization of health systems in that environment, demand for health services, patterns of morbidity and the number of health care workers (HCWs) on strike, as well as the level of participation (4).

Although strikes in the health sector have been reported on almost every continent, varying with respect to duration, demands and impact, their effects have been worse in low-and middle-income countries (LMIC) due to infrastructure and resource challenges, weak institutional arrangements, less efficient organizational ethics codes and practices, and lack of alternative available and affordable health care (1, 2). Poor and disadvantaged people report greater unmet health care needs when there are strikes (4). According to the Human Development Index (HDI), sub-Saharan Africa (SSA) is one of the poorest regions in the world, and today, with international poverty line at $1.90 per person per day, SSA accommodates the largest number of poor people in the world (5).

Various factors have been reported as the cause of strikes in several studies, ranging from wage delays, housing and risk allowances, career advancement, continuing education, deterioration of academic facilities, low numbers of doctors in training, working conditions, shortages of essential medicines as well as political oppositions related to leadership and management in health, and government economics for health (2, 6–8).

These factors lead to dissatisfaction among HCWs which in turn leads to migration, strikes and low quality of health services (9, 10). The already inadequate health systems of SSA, have been severely hampered by the shortage of human resources and the “brain drain” from Africa to Europe, the Middle East and North America: strikes further compound this problem (11, 12).

SSA countries have the most severe shortage of human resources for health (HRH) in the world, with more than 60% of countries with extreme HRH shortages found in the African region. Studies show that 47 countries in SSA have a critical shortage of HCWs, with an approximate deficit of 2.4 million doctors and nurses. These data denote a crisis in the health sector and strikes are an aggravating factor and a barrier to achieving universal health coverage, thus compromising the 2030 Agenda for Sustainable Development with respect to health (13–15).

Therefore, studies that aim to understand the causes and consequences of strikes in the health system are essential to better target actions to minimize the potential for new strikes, reduce the negative effects of strikes, if they occur, and nurture the robustness, resilience and anti-fragility of the health systems (2).

This study aims to identify the main actors, the reasons behind the industrial actions, protests, strikes and lockouts (IAPSL) in SSA countries and their impact on HCWs (specifically doctors) and health services, as well as to identify the main strategies adopted to tackle them.

## 2 Methods

### 2.1 Study design

This is a systematic review (SR) performed in accordance with the Preferred Reporting Items for Systematic reviews and Meta-Analyses (PRISMA) checklist (16–18). The study protocol for this systematic review was registered in the PROSPERO International Prospective Registry of Systematic Reviews (registration number CRD42022334173). The study was carried out between September 2022 and April 2023.

### 2.2 Inclusion and exclusion criteria

The type of publications considered were quantitative, observational (i.e., cohort, case–control, cross-sectional and ecological) and experimental studies, as well as quasi-experimental, mixed-methods and qualitative studies published between January 2000 and December 2021 in in the official languages of the Member States of the World Health Organization African Region, namely: English, French, Portuguese and Spanish. Gray literature was not included.

Eligibility criteria were developed using the PICOC criteria (19).

#### 2.2.1 Population/participants

The review included studies on HCWs and other actors involved in strikes in the health sector, such as health managers, NGOs, representatives of associations and civil society.

#### 2.2.2 Intervention(s) and exposure(s)

Only studies addressing IAPSL by HCWs were included, and studies on IAPSL by non-health care workers were not considered.

#### 2.2.3 Comparator(s)/control

We included studies that report changes in indicators (related to services, morbidity, mortality, well-being, working conditions or socio-political context) comparing the period during the IAPSL with the period before and after the IAPSL. Exclusion criteria are not applicable for this component.

#### 2.2.4 Outcome(s)

Studies that report consequences of IAPSL by HCWs for services, morbidity, mortality, well-being, working conditions, and socio-political aspects were included. Exclusion criteria are not applicable for this component.

#### 2.2.5 Context

All studies on IAPSL by HCWs in SSA were included, and studies on IAPSL by health care workers from countries outside SSA were excluded.

### 2.3 Information sources and search strategy

For this review, we used the following databases: MEDLINE, Google Scholar, Scopus, ProQuest, and Science Direct for a period between January 2000 and December 2021.

For the search algorithm, MESH terms, free text words and related terms were considered, taking into account the PICOC criteria components (19).

The search equations varied according to the operational specificities of each database, for example, ProQuest and Scopus have a location filter, so it was not necessary to include the terms *Africa south of the Sahara* or *sub-Saharan Africa* in the equation (as can be seen in Table 1).

**Table 1:** Search algorithm in different databases.

Electronic databases	Search algorithm	Filters applied	Date	Result
MEDLINE	“Africa South of the Sahara” [MeSH Terms] AND “Health personnel”[MeSH Terms] OR “healthcare workers” OR “health care worker” OR “health professionals” OR “health workforce” OR “doctor” OR “physicians”[MeSH Terms] AND “Strikes, Employee”[MeSH Terms] OR “industrial action” OR “Strikes, Employee”[MeSH Terms] OR “protest”	Time limit: January 2000–December 2021 Language: English, French, Portuguese and Spanish.	2022-08-18	1,499
ProQuest	(“healthcare workers” OR “health care worker” OR “health professionals” OR “doctor” OR “physicians”) AND (“Strikes” OR “industrial action” OR “grievances” OR “protest”)	Time limit: January 2000–December 2021 Document type: articles Location; Africa, Nigeria, Liberia, Kenya, Ethiopia, Ghana, Uganda, Zimbabwe and West Africa Source type: Scholarly Journals.	2022-08-18	899
Google scholar	“Sub-Saharan Africa” AND “Health personnel” OR “healthcare workers” OR “health care worker” OR “health professionals” OR “health workforce” OR “doctor” OR “physicians” AND “Strikes” OR “industrial action” OR “grievances” OR “protest”	Time limit: January 2000–December 2021 Language: English, French, Portuguese and Spanish.	2022-08-18	2,900
Scopus	“Health personnel” OR “healthcare workers” OR “health care worker” OR “health professionals” OR “health workforce” OR “doctor” OR “physicians” AND “Strikes” OR “industrial action” OR “grievances” OR “protest”	Time limit: January 2000–December 2021 Language: English, French, Portuguese and Spanish. Document type: article Source type: Journal Country/territory: South Africa, Nigeria, Kenya, Ethiopia, Tanzania, Cameroon, Congo, Cote d’Ivoire, Malawi, Botswana, Mozambique, Guinea-Bissau, Senegal, Uganda	2022-08-18	70
Science direct	“Sub-Saharan Africa” AND (“Health personnel” OR “healthcare workers” OR “health professionals” OR “doctor” OR “physicians”) AND (“Strikes” OR “industrial action”)	Time limit: January 2000–December 2021 Article type: Research articles Language: English, French, Portuguese, and Spanish. Content type: Journal article	2022-08-18	153

### 2.4 Data management

The electronic search was performed by the first reviewer (A.L.J.M) and the search results in each database (MEDLINE, Scopus, ProQuest and Science Direct) were directly exported to Rayyan web-tool (20), except Google Scholar, since this does not include a mechanism for exporting all results simultaneously. With the Rayyan web tool, duplicates were eliminated, and then the screening and selection of studies was performed. For results obtained through Google Scholar, screening and selection were done manually.

### 2.5 Selection of studies

The document selection process was conducted independently by two reviewers (A.L.J.M. and I.C.). In case of disagreement, a third reviewer (M.S) was consulted. First, the eligibility criteria were applied to the titles and abstracts of the identified articles, followed by the retrieval of the selected articles, and subsequently the eligibility criteria were applied for the full text screening of the retrieved articles.

### 2.6 Assessment of risk of bias

Joanna Briggs Institute (JBI) critical appraisal tools were used to assess the risk of bias of the included studies. A checklist adapted by Betran et al. was used to evaluate ecological studies (21, 22). The researchers agreed on a minimum percentage of 70% of the total items in the assessment tools to include studies in the review (21). This process was conducted independently by two reviewers (A.L.J.M. and I.C.), and in case of disagreement the third reviewer (M.S) was consulted.

### 2.7 Evidence quality assessment

The overall quality of each evidence was assessed using the Grading of Recommendations Assessment, Development and Evaluation (GRADE) approach (23), considering the limitations, inconsistency, indirectness and imprecision of the studies, and were classified as high, moderate, low or very low quality. Two reviewers (A.L.J.M. and I.C.) independently assessed the quality of evidence using GRADE, and possible discrepancies were decided through discussion or consultation with a third author (M.S).

### 2.8 Data extraction

Documents for data extraction were randomly divided between the two reviewers (A.L.J.M. and I.C.). Data were extracted using a RedCap (24) electronic data collection spreadsheet prepared according to the design of the studies involved. The extracted data comprised: characteristics of the studies (authors, year of publication, place of study, type of study and data source), population (all actors involved in the IAPSL, their profession), intervention (duration of IAPSL, causes and strategies adopted to address or mitigate its effects), comparison (comparative aspects between the period before, during and after IAPSL) and outcomes (consequences of IAPSL for services, health care workers and users). The reviewers collaborated in case of doubts or questions. The third reviewer (M.S) was consulted to resolve any discrepancies.

### 2.9 Synthesis of data

The narrative synthesis was used to review and synthesize the data extracted from the papers included in this study. It was carried out by two reviewers (A.L.J.M. and I.C.), at first individually (until the preliminary synthesis of the data) and then jointly to ensure alignment between them. The final synthesis was shared with the other authors (M.S, P.F, and A.J.R.C) for critical evaluation and identification of eventual biased interpretations. Tables and figures are used, as appropriate, to present the information, disaggregating it according to the PICOC criteria (19) to answer the research questions. Due to the nature of the questions and the objectives of the research (which aims to identify the actors involved in strike movements, all the possible causes and implications), heterogeneity and sensitivity analyses were not carried out, so meta-analysis was not performed.

### 2.10 Level of agreement among reviewers

The agreement between reviewers (A.L.J.M. and I.C.) was evaluated using the Kappa statistic, and for kappa values between 0.0–0.20 it was considered none agreement, 0.21–0.39 minimal, 0.40–0.59 weak, 0.60–0.79 moderate, 0.80–0.90 strong and above 0.90 almost perfect (25).

## 3 Results

**Figure 1:**
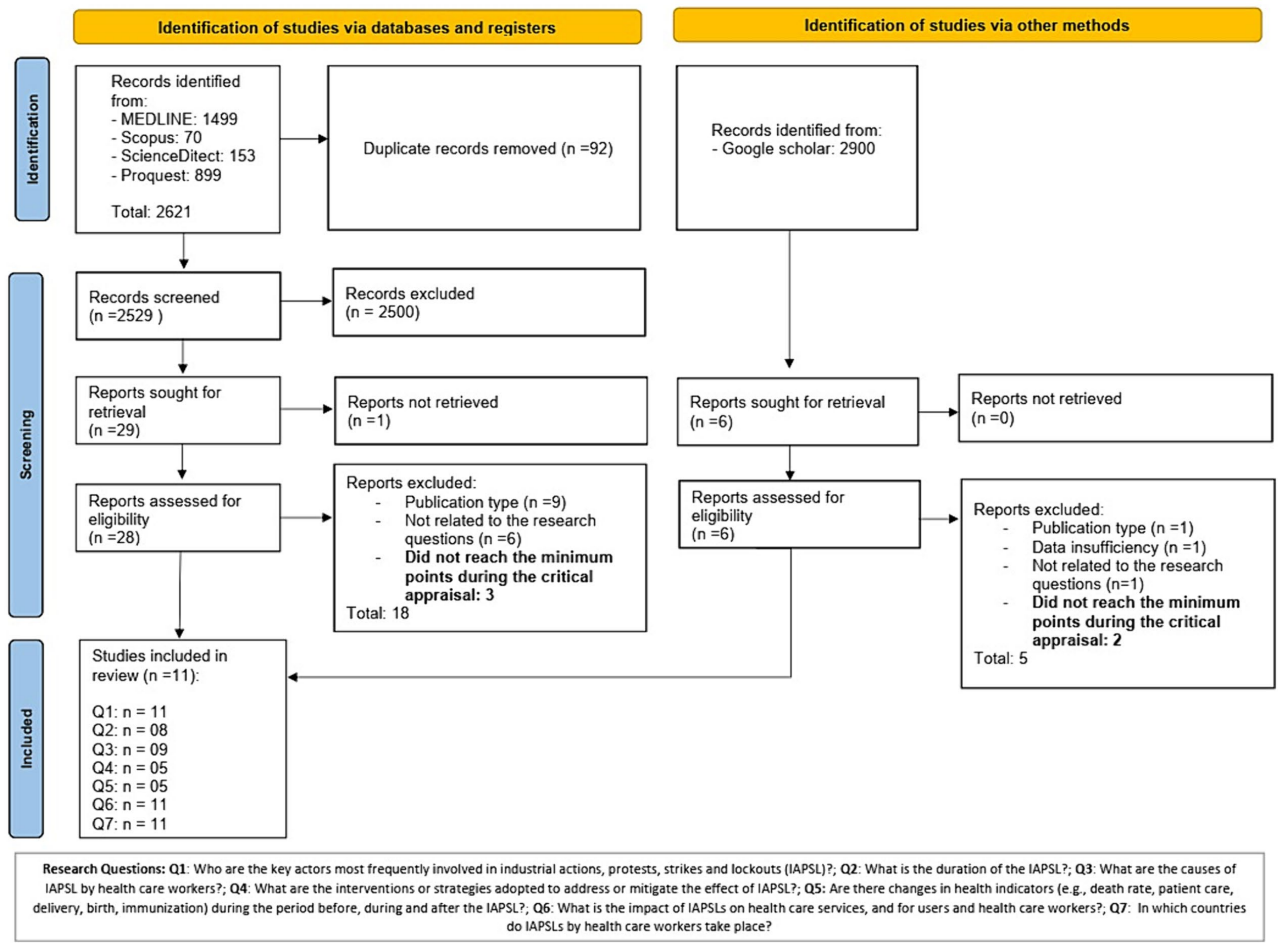
Modified version of the PRISMA 2020 flow diagram by Page et al. (17).

Through the MEDLINE, Scopus, ProQuest and Science Direct databases, 2,621 studies were identified and directly exported to the Rayyan web-tool. Duplicates were eliminated (*n* = 92) and 2,529 studies were submitted to the screening process based on title and abstract, of which 29 studies were selected for full text retrieval (Figure 1). Only 28 studies were retrieved, of which only 13 studies met the inclusion criteria and therefore underwent risk of bias assessment (as can be seen in Supplementary Table S1 for additional details). A parallel process was carried out in Google Scholar, where a total of 2,900 articles were identified and 6 studies were manually selected and retrieved, of which 3 studies met the inclusion criteria and were submitted to risk of bias assessment. Therefore, a total of 16 studies were submitted to risk of bias assessment, of which 5 did not reach the minimum points and were excluded. However, 11 studies were included for review (Figure 1). Most of the research questions were addressed by the studies included. However, the strategies or interventions adopted to mitigate the effect of the strike and comparison of health indicators between the period before, during and after the strike were addressed by less than 50% of the studies (Figure 1).

Among the studies included, two were classified by the authors as ecological, two as case reports, three as qualitative, two as cross-sectional and two as quasi-experimental studies (Table 2).

**Table 2:** characteristics of studies and quality of evidence.

Authors	Study design	JBI checklist	Betran et al checklist	GRADE
		(%)	(%)	Rating
Kaguthi et al. (4)	Ecological study	-	71.4	Low
Ong’ayo et al. (26)	Ecological study	-	85.7	Low
Adam et al. (27)	Case reports	87.5	–	Very low
Muula et al. (28)	Case reports	75.0	–	Very low
Oleribe et al. (6)	Qualitative study	80.0	–	Low
Scanlon et al. (29)	Qualitative study	90.0	–	Low
Waithaka et al. (2)	Qualitative study	90.0	–	Low
Aturak et al. (30)	Cross-sectional study	77.8	–	Very low
Oleribe et al. (31)	Cross-sectional study	77.8	–	Low
Shikuku et al. (32)	Quasi-experimental study	88.8	–	Low
Scanlon et al. (33)	Quasi-experimental study	88.8	–	Moderate

Overall, the quality of the evidence is “low” according to the GRADE assessment (Table 2). The studies had important limitations that presupposed a risk of bias. Some studies were hospital-based and did not include regional data, so the effect of the strike may be underestimated. The retrospective and longitudinal nature of some studies led to data loss or incomplete follow-up. Some studies based on interviews did not describe the characteristics of the strike (intervention) and took place later, after the strike, with a risk of memory bias.

The level of agreement between reviewers (A.L.J.M. and I.C.) during the selection of studies (Kappa = 0.87 and *p* < 0.001) was strong, while for risk of bias (Kappa = 0.652 and *p* < 0.001) and evidence quality assessment (Kappa = 0.647 and *p* < 0.001) it was moderate.

### 3.1 Study location and health care workers on strike

All studies referred to strikes with no reference to other forms of IAPSL. Most of the included studies address strikes that took place in Kenya (with 7 studies) (2, 4, 26, 27, 29, 32, 33), followed by Nigeria (with 3 studies) (6, 30, 31) and finally Malawi (with 1 study) (28), as can be seen in Table 3.

**Table 3:** Description of studies included in the review.

Author	Year	Study location	Data source	Study type	Care workers on strike	Strike duration	Causes of the strike	Strategies adopted to mitigate or solve	Comparison	Outcomes
Kaguthi et al. (4)	2020	Kenya	Database of Kenyatta National Referral Hospital, AIC Kijabe Hospital, Mbagathi Hospital and Siaya Hospital	Ecological study	Physicians, nurses and clinical officers	100-day strike of doctors (December 2016 to March 2017); 150-day strike by nurses (June 2017 to November 2017); and 20-day strike of clinical officers (September 2017 to October 2017)	The government has not fulfilled the agreement reached with the classes (doctors and nurses) to improve remuneration (salaries and allowances), human resources and equipment in health facilities, as well as funding for research.	Senior physicians (consultants) and military physicians were stationed at the national referral and teaching hospital. Services were restricted to emergencies. Elective procedures were postponed or referred to private hospitals. In peripheral government hospitals, clinical staffs who often act as a filter took over operations and cases they could not manage were possibly abandoned, especially if they could not afford transport and management in private facilities.	Period without strike and with strike	There was a reduction in the number of patients treated during the strike period. During the doctors’ strike mortality reduced considerably.
Waithaka et al. (2)	2020	Kilifi, Kenya	Interviews with frontline health managers and community representatives, review of documents and the Health Information System database.	Qualitative study	Physicians and nurses	100-day strike of doctors (December 2016 to March 2017); and 150-day strike by nurses (June 2017 to November 2017).	Failure of the government to implement the agreements signed with doctors and nurses (which established an increase in subsidies). Dissatisfaction with human resource processes and poor working conditions. Differences and injustices among health care workers. Political aspects associated with nurses and other government actors focused on national and municipal elections. Poor coordination between national and local governments affected the handling of the strike.	Prioritization of specific services (emergency and chronic care), minimizing and managing conflicts between striking and non-striking nurses, NGO staff continuing to offer services in supportive areas (such as TB/HIV), changing tasks for students and others non-strike cadres. In some facilities, support staff would have dispensed medication for minor ailments. Increased cooperation with the private sector (adoption of a system of transferring patients for post-operative care in private facilities), increased number of supervisory visits to private facilities and provision of supplies to these facilities. The community launched protest messages against the continuation of the strike.		Interruptions in service delivery (reduced hospitalizations and outpatient services), delay in accessing care, maternal complications and deaths, new-born deaths and long-term complications from delayed treatment, search for other care alternatives (private sector and traditional treatment) and increased costs of care (some families had to borrow or sell assets to pay for private services), overload and demotivation of non-striking health care workers, slower service delivery, loss of confidence in the public health sector among the community.
Scanlon et al. (29)	2021	Trans Nzoia, Kenya	Interviews with women who were pregnant during strikes in 2017, community health volunteers (CHVs) and health facility managers.	Qualitative study	Physicians, nurses and clinical officers	100-day national Physicians’ strike (December 2016 to March 2017); 44-day Trans Nzoia nurses’ strike (February 2017 to March 2017); 150-day national nurses’ strike (June 2017 to November 2017); 20-day clinical officers’ strike (September 2017 to October 2017).	Non-compliance by the government with the agreement reached with the classes (physicians and nurses) to improve remuneration (salaries and subsidies), better working conditions, delays in salaries and promotions.	Waiver of fees and hiring more staff at private (mainly religious) facilities, coordination of services, referrals and supplies between public and private facilities, and provision of services in public facilities in secret or off-premises.		Peaks in maternal and child deaths and mother-to-child HIV transmission due to decreased use of antenatal care. Limited access to maternal and child services. Pregnant women were less likely to give birth in public health facilities. Those without resources have no access to health care. Several community health volunteers described stories of using their own money to help pregnant women and mothers access care during the strike. Tension and loss of trust between the community and the health system.
Scanlon et al. (33)	2021	Trans Nzoia, Kenya	Questionnaires on women were pregnant in 2017 (year of strike) and 2018 (year without strikes)	quasi-experimental study.	Physicians, nurses and clinical officers	100-day national Physicians’ strike (December 2016 to March 2017); 44-day Trans Nzoia nurses’ strike (February 2017 to March 2017); 150-day national nurses’ strike (June 2017 to November 2017); 20-day clinical officers’ strike (September 2017 to October 2017).	Non-compliance by the government with the agreement reached with the classes (physicians and nurses) to improve remuneration (salaries and subsidies), better working conditions, delays in salaries and promotions.		Period with strike and without strike	Pregnant women attended fewer antenatal consultations during the strike period compared to the non-strike period. Lower proportion of women gives birth in health facilities during the strike period. New-borns in the strike period received their first OPV 0 vaccine significantly later compared to the non-strike period.
Adam et al. (27)	2017	Rift Valley, Kenya	Africa Inland Church-Kijabe Hospital (AICKH) database	case report study	Public sector physicians (national level), including trainees	100 days (December 2016–March 2017)	Low wages and poor working conditions	Increase beds for sick new-borns. A tracking system to establish priority admissions. The medical staff, including specialized interns, at the AICKH remained working during the strike, with the exception of government-sponsored official medical interns (*n* = 10). Clinical Officers (CO) functioning as mid-level medical outreach workers, working closely with AICKH doctors, nurses and auxiliary staff to care for as many sick children and pregnant women as possible, straining the already limited resources of the hospital infrastructure. Doctors from the Kenyan military were sent to the Kenyatta National Hospital. Physicians in management positions provided support at the referral facilities.	Before, during and after strike	Excessive demand for services, refusal of referral admissions, overload and inability to care for the sickest new-borns, women in premature labour or with high-risk pregnancies. Obstetric admissions closed intermittently. The monthly death rate has increased. In the neonatal and paediatric medical services, there was an approximate four-fold increase in deaths during the strike and an almost eight-fold increase in the paediatric surgical service. In obstetrics, there was an approximately three-fold increase in monthly maternal deaths.
Aturak, Chiegil, Ademola et al. (30)	2018	Cross River, Nigéria	Questionnaire applied to patients treated at secondary health facilities	cross-sectional study (prevalence)	Health care workers					Poor quality of health care, rising costs of health care, poor adherence to medication, high rate of referrals to private hospitals, wasted time and loss of confidence in health services, and low staff morale
Shikuku et al. (32)	2020	Busia County, Kenya	Kenya Ministry of Health reports.	quasi-experimental study	Physicians, nurses and midwives	100-day national Physicians’ strike (December 2016 to March 2017); and 150-day national nurses/midwives’ strike (June 2017 to November 2017)	Disagreements over terms of service		Physicians strike period, non-strike period and nurses and midwives strike period.	The number of patients seen in maternal and new-born care at health facilities dropped slightly during the doctors’ strike and was much lower during the nurses’ strike. During the nurses’ strike, the number of patients cared for by community midwives was higher than in health units. There was a non-significant decline in macerated stillbirths and neonatal during the nurses’ and midwives’ strike.
Oleribe et al. (31)	2016 (2015)	Abuja, Nigéria	Questionnaire applied to health care workers	cross-sectional study (prevalence)	Strikes by health care workers between 2013–2015		Poor healthcare leadership and management, demand for higher wages and salaries, infrastructure and interpersonal issues.			Disruption of patient care, high referral rates to private hospitals, loss of patient follow-up, mismanagement by alternative healers, and high private hospital costs.
Muula and Phiri (28)	2003	Blantire, Malawi		case report study	Health care workers and support staff at Malawi’s main referral hospital, Queen Elizabeth Central Hospital	14 days of strike (5 October and 19 October 2001) at hospital level.	Lack of risk subsidies, poor professional subsidies, low wages, and low housing subsidies. Comparisons were made with the judicial service, where officials are better paid.	The Council of Nurses and Midwives of Malawi and the Medical Council of Malawi threatened to take disciplinary action if their members were absent from their workplace without informing their patients or making alternative arrangements to safeguard their patients’ care. Volunteers (68 from the Red Cross and 36 nursing and medical students and teachers from the University of Malawi) provided clinical nursing and support services at QECH. The government sent armed police to guard the hospital premises. Intimidation of health care workers by community and political leaders. Negotiations between representatives of health care workers and the Ministry of Health, mediated by the Malawi Human Rights Commission. The Government has promised to meet some of the requests. Workers’ representatives demanded that suspended employees be immediately reinstated and that those transferred be returned to the QECH.		The strike resulted in the closure of nearly all of the hospital’s 1,500 beds, with the exception of the burn and orthopaedic wards. The government suspended 20 officials. Some health care workers on strike were transferred from QECH to other hospitals. There was a reduction in the number of deaths in the QECH. Education for medical students, clinical officers and nursing interns was disrupted during the strike. There was a greater demand for health care in private hospitals.
Ong’ayo et al. (26)	2019	Kilifi, Kenya	Health and Demographic Surveillance System	Population-based cohort	Nurses and physicians strike	Six nationwide health care workers’ strikes (between 2011 and 2013: total of 128 strike days). 9-day physicians’ strike (Dec 5 to 13, 2011); 15-day nurses’ strike (March 1 to 15, 2012) 22-day physicians’ strike (Sept 13 to Oct 4, 2012) 42-day nurses’ strike (Dec 3, 2012, to Jan 13, 2013) 26-day nurses’ strike (Jan 16 to Feb 11, 2013) 14-day physicians and nurses’ strike (Dec 10 to 23, 2013)			Period with strike and without strike	Service delivery was stopped, hospitalization rates reduced (paediatric hospitalization services were limited), admissions were restricted to the most critical cases. There were no significant changes in mortality between the non-strike and strike periods.
Oleribe et al. (6)	2018 (April and June 2017)	Nigéria	Questionnaire for physicians who attended the recently completed West African College of Physicians (WACP)/Royal College of Physicians (RCP) Millennium Development Goal 6 Partnership for African Clinical Training (M-PACT) course.	Qualitative study	Health care workers		The lack of well-being of care workers, salary issues, leadership and management, precarious hospital infrastructure, poor guidance and services, and disputes between care workers			Interruption in the provision of key health services, including immunization services and prevention of mother-to-child transmission of HIV, poor patient care, increased morbidity and mortality, reduced revenue generation by hospitals, loss of confidence in the health system, conflicts between staff and management, loss of dignity and respect for the profession, poor public perception of medical staff, reduced efficiency of services, distortions of patient care indicators (clinical and patient satisfaction), resident physicians are unable to timely fulfil demands for their training, poor performance in postgraduate exams with huge financial losses, Interruption of research activities.

#### 3.1.1 Kenya

Of the 7 studies, 6 addressed the HCWs’ strike in Kenya in 2017 (2, 4, 27, 29, 32, 33), and one study addressed the HCWs’ strikes that occurred in Kenya between 2011 and 2013 (26). In 2017, 4 episodes of strikes were reported: national doctors strike, national nurses strike, Trans Nzoia nurses strike and national clinical officers’ strike. Between 2011 and 2013, six national strikes episodes were reported: two doctors’ strikes, three nurses’ strikes, and one doctors’ and nurses’ strike.

#### 3.1.2 Nigeria

The three studies address the strike of HCWs (6, 30, 31), and only one specified the period of occurrence of the strikes under study (2013–2015) (31). There was no description of the number of strike episodes, much less of their duration.

#### 3.1.3 Malawi

An episode of strike by all HCWs and support staff at Malawi’s main referral hospital, Queen Elizabeth Central Hospital, which occurred in 2001, was reported (28).

**Figure 2:**
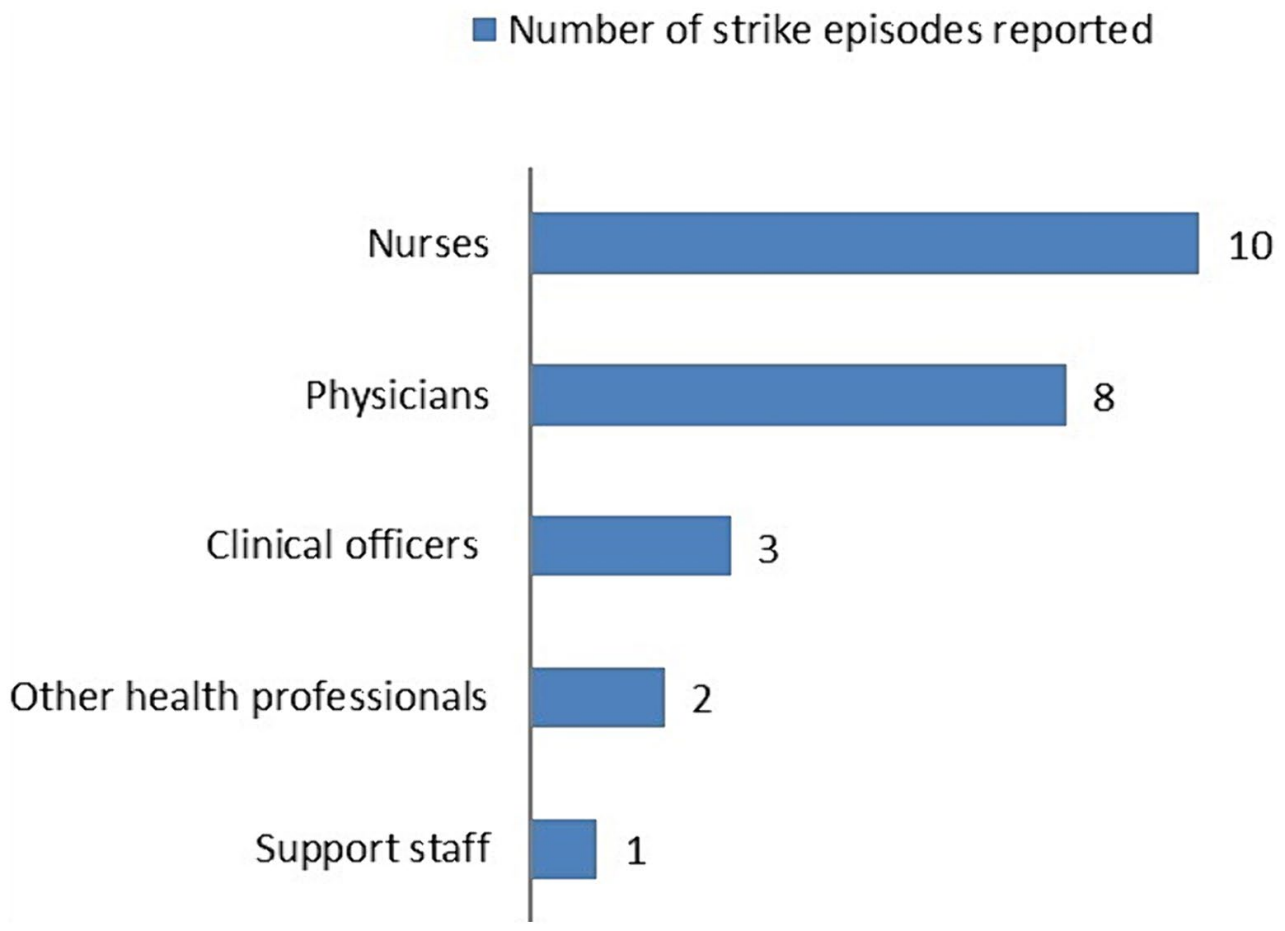
Frequency of involvement of each class of health care workers in strikes.

In general, nurses and doctors are the ones most commonly involved in strike episodes (Figure 2).

### 3.2 Duration of strikes

The duration of strikes ranged from 9 to 150 days, with an average of 41.5 days. Nurses’ strikes, on average, last longer (55.4 days) than doctors’ strikes (43.6 days).

### 3.3 Causes of strikes

The most commonly cited causes of HCWs’ strikes were disagreements regarding the salary and allowances earned by HCWs when compared to some other public servants and also poor working conditions (2, 4, 6, 27–29, 31–33). Other causes mentioned were linked to relationship with the leadership and management, interprofessional relations and dissatisfaction with political aspects (Table 3) (6, 31).

### 3.4 Strategies adopted to mitigate the effects of the strikes

To mitigate the effect of HCWs’ strikes, several measures were adopted. These vary depending on the context and the HCWs involved.

#### 3.4.1 Services provision

The most commonly adopted strategy to mitigate the effects of strikes was to restrict services and give priority to emergency conditions and chronic care, in some cases with a screening system to establish priority access/admissions (2, 4, 26, 27). Greater cooperation with the private sector was also sought (waiver of fees and hiring more staff at private facilities, provision of supplies and referral of cases for hospitalization) (2, 4, 29).

#### 3.4.2 Collaboration of non-strike actors

Other strategies adopted were the readaptation of the tasks of the available staff, such as: doctors in management positions and doctors from the military forces provided clinical support in the health care units of the striking HCWs; clinical staffs who often act as a filter took over operations; nursing and medical students and teachers provided clinical nursing and support services (2, 4, 27, 28).

### 3.5 Consequences of strikes (outcomes)

Among the studies, the most commented consequences of the strikes by HCWs were: disruption in the provision of services (with a reduction in hospitalizations and outpatient services), delays in care, higher rate of referrals to private hospitals, increase in the out of pocket costs with private services and the loss of confidence in the public health system within the community (2, 4, 6, 26–31).

The effects of the strikes on maternal and child services were the most commonly addressed, with a decrease in the use of prenatal care—pregnant women had fewer prenatal consultations—and a reduction in the proportion of women giving birth in public health care units. Peaks of mother-to-child HIV transmission, maternal complications and deaths, new-born deaths and delays in the immunization process were also reported (2, 4, 6, 27, 29, 31–33).

With regard to mortality, the majority of hospital-based studies reported a reduction in mortality (4, 28), and only one study reported an increase (27), but in the latter, a religious hospital, its employees were not on strike, with a greater inflow of patients and overload of services, which may have caused the increase in mortality. Two population-based studies that assessed mortality during the strike period did not find significant changes (26, 32).

For non-striking HCWs, there was a greater workload, weariness, and lack of motivation (2, 30). Training programs were disrupted during the strike and care workers in training (specialization) were unable to finish their training programs on time (6, 28).

For HCWs on strike, loss of dignity, respect and prestige before society were reported (6). There were reports of cases of suspension or transfer of HCWs on strike (28).

## 4 Discussion

Over the past century, strikes have been a common occurrence, across the world and among HCWs (34). The contours and consequences of the strikes includes a multitude of factors, among them: the causes, the subjects involved, the strategies adopted to solve it and the place where it occurs.

This study aimed to identify the HCWs most frequently involved in strikes, the main motivations for strikes (causes), the consequences of strikes for HCWs (strikers and non-strikers), for services and users, and the measures or strategies adopted to mitigate the effects of the strike, always in the context of SSA.

Strikes by HCWs have been a global phenomenon that affects various countries and social groups regardless of their socio-economic level (35). Dissatisfaction with salaries, allowances and working conditions have been the main reasons for strikes by HCWs around the world, and studies have shown a direct relationship between these factors and the satisfaction, motivation and productivity of HCWs and, consequently, the quality of healthcare services and the satisfaction of patients themselves (36, 37). The results show that failure to reach timely common understanding between the government and HCWs or lack of compliance on agreements achieved by the government (regarding salaries, allowances and working conditions) have been the triggers for strikes in the health sector, coinciding with the findings of Chima (in 2020), in which failed negotiations over remuneration and failure to address poor working conditions were the main cause of HCWs strikes in developed countries (such as the UK, USA, Norway, Israel and Portugal), as well as in less developed countries (such as Haiti, Uganda, Sudan, and Zimbabwe) (38).

The strikes reported in the studies included in this review occurred exclusively in the public sector. In fact, during strikes private health care units (for-profit and not-for-profit) remain in operation and maintain the healthcare services helping to overcome the service-deficit caused by the strikes. During the strikes, some interventions, such as restricting services to bare essential and prioritizing emergency cases, were the most highlighted as a way of mitigating the effects of the strike, and could even reduce including hospital mortality in the public sector, as reported in some studies (4, 28). Private for-profit health care units are less accessible to a large part of the population, since they impose costs on users which are an important barrier to healthcare services access, particularly in SSA countries where poverty is prevalent in many countries (5, 39). The demand for private non-profit health services (such as religious ones) ends up being greater due to better accessibility (low cost). Therefore, it is understandable that the impact of the strikes in these health units was associated with work overload, exhaustion and demotivation of the available staff, causing a reduction in the quality of services provided and a worsening of health indicators, such as an increase in the mortality rate (2, 27, 30).

Another strategy used to mitigate the effects of strikes was to readapt the tasks of the available staff, mainly doctors in management positions and military doctors, so that they could provide clinical support in the health care units affected by the strike (2, 30, 32), showing a low tendency for these groups to get involved in strike movements. The International Labour Organisation considers the right to strike to be restrictive or prohibitable in the case of public servants exercising authority on the state’s behalf (such as doctors in management positions) or workers in essential services whose interruption could endanger the life or safety of all or part of the population (31, 40). Military doctors belong to the defence forces, so their services are considered essential for the security of the population, and the services provided by HCWs are considered essential because they deal with life and the patient’s health is held in the highest esteem. The essence of medical practice is to save and preserve life, to promote and administer health, so it is always expected that the HCWs’ actions will always seek to preserve the patient’s health. Therefore, their rights are limited by their responsibility to save lives and promote health, according to the code of conduct for medical practice (7). On the other hand, there are those who argue that, in a democratic state, strike action is a fundamental right of workers during collective bargaining and in labour relations. It is the right of every human being to defend a fairer salary for themselves and to fight for the satisfaction of their needs, so much so that denying this right to anyone would be an act in favour of slavery (35). Some voices in the literature argue that HCWs are as essential as those who collect garbage or waste, and that uncollected garbage or unprocessed sewage is just as dangerous and has many more side effects on health than untreated pneumonia or appendicitis, and that those who advocate a strike ban must demonstrate that healthcare is as important as they say. It is understood that health care results from a joint effort and shared responsibility between HCWs, the government and society. It takes everyone’s commitment to keep the health system functional. Government and society have a responsibility to provide the necessary means for HCWs to care for patients (34). Some argue that threats to patient safety are safeguarded by providing minimum services during the strike, so HCWs should enjoy the same labor rights as other workers. Therefore, only urgent and emergency cases would be attended to, all other procedures (such as non-urgent surgeries, investigations, outpatient consultations, routine general practice consultations and documentation) would be postponed (41). However, the urgent nature of a clinical condition sometimes carries a certain subjectivity, and the fact is that if the patient comes to the health unit it is because they have concerns, so there is a need to understand how patient feels and understand the postponement of his care. Therefore, studies that assess patients’ perceptions of the HCWs’ strike and its implications are important to understand the problem from different perspectives.

However, strikes tend to undermine interprofessional relationships and those between managers and other HCWs, bringing mistrust and conflict within work teams. In turn, labour conflicts can affect the productivity and motivation of HCWs, limiting their contributions, altering the dynamics and communication in work teams, affecting the efficiency of the services provided to patients (42, 43). Strikes also lead to a loss of trust in the healthcare system and doctors in society, which can be associated with a decrease in hospital adherence, a worsening of patients’ health status, a decrease in adherence to medication and medical recommendations, and low overall satisfaction with healthcare on the among patients (39, 44).

As a result of strikes, there are also interruptions in medical training (undergraduate and postgraduate), some doctors were suspended and others transferred, meaning a setback for the national health system, further aggravating the human resources for health (HRH) crisis in this region (SSA), which had a shortage of approximately 2.4 million doctors and nurses (13–15). Another aggravating factor for this (HRH) crisis is the brain drain (emigration) from LMIC to high-income countries due to the exhaustion, demotivation and disillusionment generated by the strikes in the health sector (45, 46).

During strikes, there are reductions in prenatal consultations and births in public health units, as well as delays in the immunization process, which represent a setback in the fight against neonatal and maternal mortality and in the prevention of vertical infections of the human immunodeficiency virus (HIV), which has been on the rise in this region (47–49), and a setback in achieving the Sustainable Development Goals (specifically the third) defined by the United Nations, which have maternal and child health indicators as a priority (50).

### 4.1 Study limitations

The study was limited in time (2000–2021) and in space (SSA) and intended to approach this phenomenon within a current and particular context which, although endowed with a certain cultural, socio-political and economic diversity, as is the case of SSA, may share needs and challenges in this part of African continent (51).

This review included peer-reviewed studies and excluded grey literature and additionally excluded a total of five studies for not achieving the minimum quality required for the review which, somehow, restricted its results to strikes in Kenya, Nigeria and Malawi. Therefore, these results cannot be fully extrapolated across SSA because there was not much diversity with respect to the study location (countries).

Among SSA countries, Kenya is one of best ranked in terms of income (in the list of Lower-Middle Income Economies Countries) (52) and HDI (0.575, in position 152) (53). Therefore, the consequences of strikes may be worse in other SSA countries, due to greater scarcity of resources, lack of available and accessible health care alternatives and unmet health needs (1, 2, 4).

According to the systematic review carried out by Russo et al. (9), there have been many strike episodes among SSA countries in the last two decades, but few studies have been published in peer-reviewed journals, and unlike this study, the review by Russo et al. (9) included grey literature (and countries beyond SSA). The inclusion of grey literature in the systematic review is a challenge given the difficulty in analysing the credibility and quality of the information, since the sources of information are not always referenced, which jeopardizes the quality of the review (54). Although, on the other hand, its inclusion may reduce the risk of publication bias and provide more balanced results, since many studies are not published in peer-reviewed journals for reasons such as the complexity of the submission process, long waiting time until publication, high rejection rates or even when the results are null or negative (55).

On the other hand, the studies included are lacking in terms of quality (in general, they have low quality according to GRADE) and lacked a detailed description of the strike and its contours, such as, for example, the strategies adopted to stop the strike, the proportion of non-striking HCWs and the socio-political or epidemiological context in which they occur.

However, the reality of private for-profit health units during the strike period was little known, and this information should be on the agenda since, according to Yoong et al. (56), the participation of the private sector in SSA is positively associated with better functioning of the health system in terms of access and equity. It is suggestive to think that the effect of the HCWs’ strikes in the public sector might be worse without the active participation of the private sector in providing substitution services. Another fact to consider is the role that the traditional medicine has played in health care, especially in SSA, where, for example, in countries like South Africa, 80% of the population seek health care from traditional healers (57). Despite this reality, the demand for services provided by traditional healers was not discussed in these studies. Therefore, future studies that address the impact of strikes by HCWs should consider not only the private sector but also the traditional health services.

Most of the included studies do not address the resolution of the strike, what happened to stop the strike and consequences or overall outcomes after the strike. The resolution of strikes is not always peaceful and pleasant for parties involved, as was the case with the strike in Malawi (in 2001), where as a result, HCWs were transferred, suspended, dismissed and the government’s promises were not kept (28). These actions are probably not the most appropriate way to resolve the strike problem, as they could lead to legal action and disputes between the parties, causing discomfort, mistrust, resentment and further distancing between employees and employers, producing an effect contrary to the desired one (35). These conflicts, if unresolved, set precedents with an impact on the future, subsidising future strikes and leaving historical marks that are difficult to overcome.

## 5 Conclusion

Among HCWs, nurses and doctors are the most involved in strike movements, and aspects associated with salaries and working conditions have dominated the grievances’ agendas and have stood out as the main causes of strikes in the healthcare sector in SSA. Strikes occurred in the public health sector and, to mitigate their impact, the most prominent strategy was to restrict health services and prioritize emergency cases, causing greater demand for private services and worsening healthcare costs. To make up for the shortage of human resources, the tasks of the available staff have been readapted, such as military doctors and those with management positions who have been helping in the health units, but there has been overload, tiredness, and demotivation among the available staff. The strikes compromise access to health care systems and the well-being of patients, especially the most disadvantaged. They may represent a setback for a country’s development, with implications for everyone involved in the health system. Therefore, there is a need to adopt strategies to prevent this phenomenon or timely resolution through regulation and negotiation to balance the rights of HCWs and the rights of patients.

## Data availability statement

The raw data supporting the conclusions of this article will be made available by the authors, without undue reservation.

## Author contributions

AM, IC, and PF worked on designing the study protocol. AM, IC, and MS conducted the systematic literature review. AM worked on the conceptualization and writing of the original draft. IC, PF, AC, and MS reviewed and edited the manuscript. All authors contributed to the article and approved the submitted version.
