# Supplementary material for: Strikes of physicians and other health care workers in sub-Saharan African countries: a systematic review
Source: Front Public Health. 2024 May 30;12:1209201. doi: 10.3389/fpubh.2024.1209201 (PMC11169935; doi:10.3389/fpubh.2024.1209201)
Supplement: Supplementary file 1 [file Table_1.DOCX]

Supplementary Material

# Supplementary Tables

Table 1: Characteristics of all studies retrieved during the systematic review

| **Decision** | **Reason for exclusion** | **Authors (year of publication)** | **Title** | **Publication type** | **Country** | **DOI** |
| --- | --- | --- | --- | --- | --- | --- |
| Excluded | publication type | Dhai and Mahomed (2018) | The National Education, Health and Allied Workers’ Union (NEHAWU) strikes: South Africa’s healthcare battlefield | Reflection | South Africa | 10.7196/SAMJ.2018.v108i8.13458 |
| Included |  | Kaguthi, Nduba and Adam (2020) | The impact of the nurses’, doctors’ and clinical officer strikes on mortality in four health facilities in Kenya | Original article | Kenya | 10.1186/s12913-020-05337-9 |
| Excluded | publication type | Alubo and Hunduh (2017) | Medical Dominance and Resistance in Nigeria’s Health Care System | Review | Nigeria | 10.1177/0020731416675981 |
| Included |  | Waithaka, et al. (2020) | Prolonged health worker strikes in Kenya perspectives and experiences of frontline health managers and local communities in Kilifi County | Original article | Kenya | 10.1186/s12939-020-1131-y |
| Included |  | Shikuku, et al (2020) | The effect of the community midwifery model on maternal and newborn health service utilization and outcomes in Busia County of Kenya: a quasi-experimental study | Original article | Kenya | 10.1186/s12884-020-03405-w |
| Excluded | Not related to the research questions: | Rensburg, A. and Rensburg, D. (2013) | Nurses, industrial action and ethics: Considerations from the 2010 South African public-sector strike | Review | South Africa | 10.1177/0969733012473771 |
| Included |  | Oleribe, et al.(2016) | Industrial action by healthcare workers in Nigeria in 2013–2015: an inquiry into causes, consequences and control—a cross-sectional descriptive study | Original article | Nigeria | 10.1186/s12960-016-0142-7 |
| Excluded | Did not reach the minimum points during the critical appraisal | Njuguna (2018) | Impact of Nurses' Strike in Kenya on Number of Fully Immunized Infants in 18 County Referral Hospitals | Original article | Kenya | 10.1353/hpu.2018.0095 |
| Excluded | Not related to the research questions: | Muller (2001) | Strike action by nurses in South Africa: A value clarification | Original article | South Africa | 10.4102/curationis.v24i4.884 |
| Excluded | Did not reach the minimum points during the critical appraisal | Bhuiyan and Machowski (2012) | Impact of 20-day strike in Polokwane Hospital  (18 August - 6 September 2010) | Original article | South Africa | 10.7196/samj.6045 |
| Included |  | Muula and Phiri (2003) | Reflections on the health workers' strike at Malawi's Major Tertiary Hospital, QECH, Blantyre, 2001: a case study | Original article | Malawi | 10.1191/0969733003ne595oa |
| Excluded | Not related to the research questions: | Akinyemi and Atilola (2012) | Nigerian resident doctors on strike: insights from and policy implications of job satisfaction among resident doctors in a Nigerian teaching hospital | Original article | Nigeria | 10.1002/hpm.2141 |
| Excluded | Not related to the research questions: | Mayaki and Stewart (2020) | Teamwork, Professional Identities, Conflict, and  Industrial Action in Nigerian Healthcare | Original article | Nigeria | 10.2147/JMDH.S267116 |
| Included |  | Scanlon, et al. (2021) | ‘It was hell in the community’: a qualitative study of maternal and child health care during health care worker strikes in Kenya | Original article | Kenya | 10.1186/s12939-021-01549-5 |
| Excluded | Not related to the research questions: | Aborisade and Gbahabo (2021) | Policing the lockdown: accounts of police officers’ aggression and extortion of frontline health workers in Nigeria | Original article | Nigeria | 10.1080/10439463.2021.1903461 |
| Excluded | publication type | Gathongo and Ndimurwimo (2020) | Strikes in Essential Services in Kenya: The Doctors, Nurses and Clinical Officers' Strikes Revisited and Lessons from South Africa | Reflection | South Africa | 10.17159/17273781/2020/v23i0a5709 |
| Excluded | publication type | Ravez, et al. (2019) | Les grèves de médecins en République Démocratique du Congo : quels repères éthiques généralisables? | Reflection | Democratic Republic of Congo | 10.7202/1062303ar |
| Included |  | Oleribe, et al. (2018) | Healthcare workers’ industrial action in Nigeria: a cross-sectional survey of Nigerian physicians | Original article | Nigeria | 10.1186/s12960-018-0322-8 |
| Excluded | data insufficiency | Okontaa, K. and Okontac, O. (2017) | Industrial crises in a Tertiary Health Institution (THI) in Nigeria: The perspective of resident doctors | Original article | Nigeria | 10.1080/20479700.2017.1397323 |
| Excluded | publication type | McQuoid-Mason and Bcomm (2018) | What should doctors and healthcare staff do when industrial action jeopardises the lives and health of patients? | Reflection | South Africa | 10.7196/SAMJ.2018.v108i8.13479 |
| Excluded | Not related to the research questions: | Munyaradzi (2010) | Are physicians’ strikes ever morally justifiable? A call for a return to tradition | Original article | Mozambique | 10.4314/pamj.v6i1.69081 |
| Excluded | publication type | Ogunbanjo and Bogaert (2009) | Doctors and strike action: Can this be morally justifiable? | Reflection | South Africa | 10.1080/20786204.2009.10873869 |
| Included |  | Ong’ayo, et al. (2019) | Effect of strikes by health workers on mortality between 2010 and 2016 in Kilifi, Kenya: a population-based cohort analysis | Original article | Kenya | 10.1016/S2214-109X(19)30188-3 |
| Excluded | publication type | Das (2021) | Cancer care crisis in Nigeria amid doctors’ strike | News |  | 10.1016/S1470-2045(21)00569-6 |
| Included |  | Scanlon, et al. (2021) | A retrospective study of the impact of health worker strikes on maternal and child health care utilization in western Kenya | Original article | Kenya | 10.1186/s12913-021-06939-7 |
| Excluded | publication type | Makoni (2019) | Doctor strikes in Zimbabwe: fighting for provision of health | News | Zimbabwe | 10.1016/S0140-6736(19)30198-9 |
| Excluded | publication type | Irimu, et al. (2018) | Tackling health professionals’ strikes: an essential part of health system strengthening in Kenya | Commentary | Kenya | 10.1136/bmjgh-2018-001136 |
| Included |  | Adam, et al. (2018) | Paediatric and obstetric outcomes at a faith-based hospital during the 100-day public sector physician strike in Kenya | Original article | Kenya | 10.1136/bmjgh-2017-000665 |
| Excluded | Did not reach the minimum points during the critical appraisal | Njuguna (2015) | Impact of Health Workers’ Strike in August 2014 on Health Services in Mombasa County Referral Hospital, Kenya | Original article | Kenya | 10.1353/hpu.2015.0106 |
| Excluded | Not related to the research questions: | Dhai, et al.(2011) | The public’s attitude towards strike action by healthcare workers and health services in South Africa | Original article | South Africa | <https://www.ajol.info/index.php/sajbl/article/view/72986> |
| Excluded | Did not reach the minimum points during the critical appraisal | Nyango and Mutihir (2021) | Trend of health worker’s strike at a Tertiary Health Institution in North Central Nigeria | Original article | Nigeria | 10.25259/ANMRP_9_2020 |
| Excluded | publication type | Osakede and Ijimakinwa (2014) | The effect of public sector health care workers strike: Nigeria experience | Reflection | Nigeria | [www.arabianjbmr.com/RPAM_index.php](http://www.arabianjbmr.com/RPAM_index.php) |
| Excluded | Did not reach the minimum points during the critical appraisal | Essien (2018) | The Socio-Economic Effects of Medical Unions Strikes on the Health Sector of Akwa Ibom State of Nigeria | Original article | Nigeria | 10.18034/abr.v8i2.157 |
| Included |  | Aturaka, et al. (2018) | Effect of Health Workers Strikes on Quality of Care in Health Institution in Cross River State, Nigeria | Original article | Nigeria | 10.21522/TIJAR.2014.05.02.Art001 |
